# Analysis of the clinical characteristics of bone metastases after pheochromocytoma/paraganglioma surgery

**DOI:** 10.3389/fendo.2025.1657202

**Published:** 2025-11-11

**Authors:** Longmin Li, Hongbo Gao, Yujun Shao, Xiayang Zhu

**Affiliations:** 1 Department of Nuclear Medicine Center, Beijing Nuclear Industry Hospital, Beijing, China; 2 Nuclear Medical Molecular Targeting Research Branch Center of Chinese Nuclear Industry, Beijing, China

**Keywords:** pheochromocytoma, paraganglioma, bone metastases, clinical characteristics, prognosis

## Abstract

**Background:**

Pheochromocytomas and paragangliomas (PPGLs) frequently metastasize to bone. We aimed to investigate the clinical characteristics of PPGL patients with postoperative bone metastases (BMs) in hopes of providing valuable insights into future clinical practices for patients with PPGLs.

**Methods:**

In this retrospective study, 107 patients were enrolled. The clinical, pathological and laboratory examination results of the patients were analyzed. We statistically investigated the clinical characteristics of BMs in PPGLs. Logistic regression analysis was used to determine the factors influencing BMs, and a Cox proportional hazards regression model was used to evaluate the prognostic factors of bone metastasis-free survival (BMFS) in patients with PPGLs.

**Results:**

Eighty-one of the 107 patients (75.7%) developed BMs, and BMs occurred at a median of 60 months after complete resection of the primary mass (range, 3–308 months). Most bone lesions were multiple (54 patients [66.7%]), and the spine was the most frequent site of bone involvement (229 lesions [63.3%]). Skeletal complications occurred in 31 patients (38.3%), of whom bone pain was the most common symptom (90.3%). Fifty-eight patients were grouped according to whether they had bone metastases or not. Logistic regression analysis revealed that SDHB mutation (OR*=*5.334, 95% CI: 1.072–26.534*, P=*0.041) was an independent risk factor for bone metastases in PPGLs. The median duration of bone metastasis-free survival in the 58 patients with PPGLs was 101 months (range, 3–308 months). Multivariate Cox regression analysis revealed that SDHB mutation (HR=3.376, 95% CI: 1.470–7.754, *P*=0.004) was an independent poor prognostic factor for BMFS in patients with PPGLs.

**Conclusions:**

PPGLs exhibit a pronounced tropism for bone, metastasizing to the skeletal system with higher frequency than to organs like the liver or lungs. These bone metastases are frequently associated with skeletal complications. SDHB mutation is a risk factor for bone metastases in PPGLs. It is crucial to emphasize genetic detection and postoperative follow-up examinations in patients with PPGLs.

## 1 Introduction

In the traditional classification, tumors occurring in the adrenal medulla and extra-adrenal sympathetic and parasympathetic nervous system chains are referred to as pheochromocytomas (PCCs) and paragangliomas (PGLs), respectively. Clinically, they are collectively known as PPGLs. However, with a deeper understanding of PPGLs, the concepts of PCCs and PGLs are gradually being unified, and PCCs are classified as internal adrenal paragangliomas. PPGLs are defined as potentially metastatic because of their ability to metastasize even years after complete resection of the primary mass, posing potential challenges in disease management (1–3). It has been reported that the metastasis rate of PPGLs is up to 26% with an incidence < 1/1,000,000/year (4). In clinical practice, metastatic lesions are commonly found in bones, as well as in lymph nodes, lungs, and liver. Bone metastases can lead to various complications that significantly affect the quality of life of patients with PPGLs (5–7).

There is a lack of reliable research or long-term follow-up for postoperative BMs of PPGLs at present. The purpose of this retrospective study was to investigate the clinical characteristics of BMs in PPGLs. The secondary objectives were to evaluate factors that can predict BM occurrence and assess prognosis in patients with bone-metastatic PPGLs.

## 2 Patients and methods

### 2.1 Study population

In this retrospective study, we analyzed 107 consecutive patients referred to Beijing Nuclear Industry Hospital between 2015 and 2025 who were suspected of having postoperative recurrent or distant metastatic disease and were scheduled to undergo 131I-MIBG diagnosis and treatment at our institution. Among these patients, 81 were diagnosed with postoperative BMs. The inclusion criteria for patients with PPGL were as follows: (1) All patients had undergone surgery and were diagnosed on the basis of tumor histopathology with PPGL. (2) Regular postoperative follow-up was conducted after complete resection of the primary tumor. The clinical diagnosis of the first bone metastasis (BM) was made by a comprehensive evaluation of the patient’s medical history and imaging examinations. The exclusion criteria for PPGL patients were as follows: (1) Patients had not undergone surgical treatment. (2) Preoperative examination indicated the presence of BM. (3) Patients had bone invasion by contiguity that did not originate from bone itself or was combined with other malignant tumors.

The Ethics Committee of Beijing Nuclear Industry Hospital approved the study, which was also conducted in accordance with the provisions of the Declaration of Helsinki. The requirement for informed consent was waived because of the retrospective nature of the study, and all the data were anonymized and deidentified.

### 2.2 Clinical assessment

Patient data collected for analysis included sex, age at diagnosis, primary tumor site and size, Ki-67 index, date of initial diagnosis of BM (BMs were defined as bone lesions detected 3 months after complete resection of the primary mass), skeletal sites of BM, hormonal status at initial diagnosis of BM [24-hour urine catecholamine including norepinephrine (NE), epinephrine (E) and dopamine (DA)], skeletal complications (pain owing to BMs, fracture, spinal cord compression, hypercalcemia), bone metastasis-free survival (BMFS) and overall survival (OS), and presence of a genetic mutation.

Germline mutations were screened by using next-generation sequencing (NGS). All genetic tests were performed using one customized NGS panel comprising SDHA, SDHB, SDHC, SDHD, SDHAF2, VHL, EPAS1, EGLN1, EGLN2, IDH1, FH, MDH2, NF1, MAX, RET, TMEM127, FGFR1, HRAS and CSDE1 mutations. Mutations were confirmed by Sanger sequencing and tested in the peripheral blood of 58 patients whose blood samples were available.

Two professional nuclear medicine physicians, each with more than 10 years of experience, independently reviewed all the image reports in a double-blind manner. In cases of initial discrepancy in their interpretation, a consensus was reached through further joint discussion and by referring to the patient’s clinical history, combined with serial imaging and follow-up results spanning at least 6 months. This process ensured a unified and accurate assessment for the study.

### 2.3 Statistical analysis

BMFS duration was defined as the interval from the date of primary tumor resection to the date of initial diagnosis of BMs. OS duration was defined as the interval from the date of primary tumor resection to the date of death or the last known survival date.

All the statistical analyses were performed using SPSS 26.0. Descriptive statistics were used for patient demographics and PPGL and BM characteristics. Categorized variables are expressed as percentages. Continuous values are reported as the mean ± standard or median (25th, 75th percentiles). Continuous variables were compared by the t test or Mann–Whitney U test, and categorical variables were compared by the chi–square test or Fisher’s exact test. The parameters associated with BMs in the univariable analysis (*P*<0.05) were included in the multivariable logistic regression analysis. Survival curves were created using the Kaplan–Meier method, and differences in terms of BMFS were compared using a log-rank test. A Cox proportional hazards regression model was employed to determine hazard ratios (HRs) and 95% CIs in both univariable and multivariable analyses, with the lowest risk group used as the reference group. The parameters associated with BMFS in the univariable analysis (*P*<0.05) were included in the multivariable analysis.

Patients with missing data were excluded from the analyses if their files did not contain data for the required variables, and *P* values less than 0.05 were considered significant.

## 3 Results

### 3.1 Characteristics of patients

In this study, the median age at diagnosis among the 107 patients with PPGLs was 39 years (range, 12–76 years), with 53 (49.5%) males and 54 (50.5%) females. Among the tumors, 59 (55.1%) were PCCs, and 48 (44.9%) were extra-adrenal paragangliomas (EAPGLs); most of the tumors occurred in the unilateral adrenal and retroperitoneum. Among the 107 patients, all metastases were identified during postoperative follow-up. The most frequent site was bone (81 patients, 75.7%), followed by lymph node (54, 50.5%), liver (27, 25.2%), and lung (26, 24.3%). Postoperative pathology results indicated that the median primary tumor size of the 107 PPGLs was 6.3cm (range, 2~19 cm), 75 (70.1%) of which had a Ki-67 index of 3% or greater. Eighty-three of the 107 patients (77.6%) reported 24-h urinary catecholamine hypersecretion, of which NE was the most frequent hormone (67 patients [81.0%]) (**Table 1**).

**Table 1:** Patient characteristics.

Characteristic	No. (%)
Sex	
Male	53 (49.5)
Female	54 (50.5)
Median age at PPGL diagnosis years (range)	39 (12-76)
PCC	59 (55.1)
Unilateral	58 (98.3)
Bilateral	1 (1.7)
EAPGL	48 (44.9)
Neck	1 (0.9)
Chest	1 (0.9)
Abdomen and/or retroperitoneum	40 (37.4)
Pelvis (including bladder)	6 (5.6)
Median size of primary tumor, cm (range) *	6.3 (2-19)
Ki-67 Index (%)	
<3	32 (29.9)
≥3	75 (70.1)
Bone metastases	81 (75.7)
Lymph node metastases	54 (50.5)
Lung metastases	26 (24.3)
Liver metastases	27 (25.2)
Other metastases (Abdominal wall)	3 (2.8)
Catecholamine hypersecretion (83 patients)	
NE	67 (81.0)
E	53 (63.9)
DA	24 (28.9)

PCC represents pheochromocytoma; EAPGL represents extra-adrenal pheochromocytoma; NE represents 24-h urinary norepinephrine (normal range: 16.7~40.7 µg/24h); E represents 24-h urinary epinephrine (normal range: 1.7~6.4 µg/24h); and DA represents 24-h urinary dopamine (normal range: 120.9~330.6 µg/24h). *In cases of multiple tumor foci, the largest diameter was used for analysis.

In addition, physicians performed genetic testing in 58 patients, 34 (58.6%) were PCCs, and 24 (41.4%) were EAPGLs; 31 (53.4%) had gene mutations, 23 of which (74.2%) were in the SDHB gene, whereas 1 patient (3.2%), 1 patient (3.2%), 1 patient (3.2%), 4 patients (12.9%) and 1 patient (3.2%) had mutations in the SDHA, SDHD, VHL, RET and NF1 genes, respectively. The mutation rate of SDHB was significantly different between the PCC and EAPGL groups (*P*=0.004) (**Table 2**).

**Table 2:** Distribution of genetic mutations in PCC and EAPGL patients.

Mutations, n (%)	Total (N=31)	PCC (N=13)	EAPGL (N=18)
SDHA, n (%)	1 (3.2%)	1 (7.7%)	0
SDHB, n (%)	23 (74.2%)	6 (46.2%)	17 (94.4%)
SDHD, n (%)	1 (3.2%)	1 (7.7%)	0
RET, n (%)	4 (12.9%)	3 (23.1%)	1 (5.6%)
VHL, n (%)	1 (3.2%)	1 (7.7%)	0
NF1, n (%)	1 (3.2%)	1 (7.7%)	0

PCC represents pheochromocytoma; EAPGL represents extra-adrenal pheochromocytoma.

### 3.2 Characteristics of bone metastases

The imaging techniques most frequently used to diagnose BMs were computed tomography, magnetic resonance imaging, FDG-positron emission tomography and I-131-MIBG scintigraphy. The median duration of BMs was 60 months (range, 3~308 months), and 61 of the 81 PPGLs (75.3%) were diagnosed with BMs more than 5 years after surgery.

Among the 81 PPGL patients with bone-metastasis, metastatic disease was limited to bone in 31 patients (38.3%), whereas 27 (33.3%), 19 (23.5%) and 23 (28.4%) patients had concomitant metastases in the lymph node, liver and lung, respectively. Among the 81 PPGL patients, 17 (21.0%) had a single BM, while 64 (79.0%) had multiple (≥ 2 sites) BMs. In terms of the site of the BMs, 1 patient (1.2%) had only skull, 4 (4.9%) had only limbs, and 76 (93.8%) had diffuse bone metastases. At the time of the BM diagnosis, 69 of the 81 patients for whom data were available had a total of 362 bone metastasis lesions, 12 (3.3%) in the skull, and 17 (4.7%) in the limbs. Most (333 lesions [92.0%]) bone lesions were in the ribs, sternum, spine, pelvis, scapula and clavicle, 229 of which (63.3%) were in the spine. Thirty-six of the 81 (44.4%) patients developed skeletal complications, 30 (83.3%) patients reported pain owing to BMs, 9 (25.0%) patients had spinal cord compression, 5 (13.9%) patients had pathological fractures, and 2 (5.6%) patients had hypercalcemia (**Table 3**).

**Table 3:** Characteristics of bone metastases.

Characteristic	No. (%)
Median time of BM from primary tumor resection, months (range)	60 (3-308)
Bone metastases	
Only bone metastases	31 (38.3)
Concomitant metastases in other sites	50 (61.7)
Number of BM Sites	
1	17 (21.0)
≥2	64 (79.0)
Site of bone involvement	
Only skull	1 (1.2)
Only lower or upper limb	4 (4.9)
Diffuse bone metastases	76 (93.8)
Distribution of 362 bone metastases lesions	
Skull	12 (3.3)
Lower or upper limb	17 (4.7)
Ribs/sternum/scapula/clavicle	37 (10.2)
Spine	229 (63.3)
Pelvis	67 (18.5)
Complications related to BMs (36 patients)	
Bone pain	30 (83.3)
Spinal cord compression	9 (25.0)
Fracture	5 (13.9)
Hypercalcemia	2 (5.6)

BMs represent bone metastases.

In addition, the distributions of bone metastases in the skull, trunk bones, and limb bones did not significantly differ between the PCC and EAPGL groups (*P*=0.464) (**Table 4**).

**Table 4:** Distribution of bone metastases between the PCC and EAPGL groups.

Sites, n (%)	Total (N=362)	PCCs (N=204)	EAPGLs (N=158)
Skull, n (%)	12 (3.3%)	5 (2.5%)	7 (4.4%)
Truncal bones, n (%)	333 (92.0%)	188 (92.2%)	145 (91.8%)
Spine, n (%)	229 (68.8%)	133 (70.7%)	96 (66.2%)
Cervical vertebras, n (%)	21 (9.2%)	16 (12.0%)	5 (5.2%)
Thoracic vertebras, n (%)	106 (46.3%)	58 (43.6%)	48 (50.0%)
Lumbar vertebras, n (%)	74 (32.3%)	41 (30.8%)	33 (34.4%)
Sacrococcyx, n (%)	28 (12.2%)	18 (13.5%)	10 (10.4%)
Pelvis, n (%)	67 (20.1%)	34 (18.1%)	33 (22.8%)
Others *, n (%)	37 (11.1%)	21 (11.2%)	16 (11.0%)
Limbs, n (%)	17 (4.7%)	11 (5.4%)	6 (3.8%)

PCC represents pheochromocytoma; EAPGL represents extra-adrenal pheochromocytoma; *, includes the collarbones, ribs, sternum and scapula.

### 3.3 Risk factors for bone metastases

The 58 patients with available genetic data were divided into two groups: 32 patients with BMs and 26 patients without BMs. We analyzed the associations of sex, age ​at diagnosis, primary tumor site and size, the Ki-67 index, catecholamine status at initial diagnosis of BM, and SDHB mutation with BMs. Compared with that in the PPGLs without BMs group, the proportion of BMs in the EAPGL group was greater (79.2% vs. 38.2%, *p*=0.002), and patients with SDHB mutations were more frequently found in the BMs group (87.0% vs. 13.0%, *p*=0.000). The 24-h urinary norepinephrine (24hU-NE), 24-h urinary epinephrine (24hU-E) and 24-h urinary dopamine levels were higher in patients without BMs (*p*=0.002, *p*=0.008, and *p*=0.005, respectively). There was no significant difference in sex, age at diagnosis, primary tumor size, or the Ki-67 index between the groups (*p*>0.05) (**Table 5**).

**Table 5:** Comparison of the clinical characteristics of 58 PPGL patients with or without bone metastasis.

variable	BMs (n=32)	Without BMs (n=26)	*P* value
Sex			0.684
Male	14 (58.3)	10 (41.7)	
Female	18 (52.9)	16 (47.1)	
Age at diagnosis (years)	32.8 ± 12.7	33.5 ± 13.0	0.826
Primary tumor sites			0.002
PCC	13 (38.2)	21 (61.8)	
EAPGL	19 (79.2)	5 (20.8)	
Primary tumor size (cm)	6 (5.0, 8.3)	5.3 (5.0, 7.2)	0.474
Ki-67(%)			0.404
≥3	23 (59.0)	16 (41.0)	
<3	9 (47.4)	10 (52.6)	
NE (μg/24h)	71.6 (20.3, 133.0)	322.0 (80.8, 517.1)	0.002
E (μg/24h)	8.3 (2.6, 14.1)	19.6 (6.2, 55.1)	0.008
DA (μg/24h)	135.1 (27.9, 335.5)	314.7 (193.8, 406.0)	0.005
SDHB mutation			0.000
Yes	20 (87.0)	3 (13.0)	
No	12 (34.3)	23 (34.3)	

PCC represents pheochromocytoma; EAPGL represents extra-adrenal pheochromocytoma; NE represents 24-h urinary norepinephrine (normal range: 16.7~40.7 µg/24h); E represents 24-h urinary epinephrine (normal range: 1.7~6.4 µg/24h); DA represents 24-hour urinary dopamine (normal range: 120.9~330.6 µg/24h); and SDHB represents succinate dehydrogenase subunit B.

Logistic regression analysis was used to identify risk factors for BM in PPGLs. Univariable analysis revealed that EAPGL (OR, 3.800 [95% CI, 1.419–10.177]; *P=*0.008), lower 24h U-NE concentrations (OR, 0.997 [95% CI, 0.994–0.999]; *P=*0.013), lower 24h U-E concentrations (OR, 0.966 [95% CI, 0.935–0.998]; *P=*0.035), and SDHB mutation (OR, 12.778 [95% CI, 3.151–51.811]; *P=*0.000) were risk factors. Multivariate logistic regression analysis revealed that SDHB mutation (OR, 5.334 [95% CI, 1.072–26.534]; *P=*0.041) was an independent risk factor for BM in PPGLs (**Table 6**).

**Table 6:** Univariate and multivariate logistic regression analyses of bone metastasis.

Variable	Univariable		Multivariable	
	*OR (95% CI)*	*P*	*OR (95% CI)*	*P*
Male	1.400 (0.622-3.152)	0.416		
Age	1.005 (0.990-1.020)	0.512		
EAPGL	3.800 (1.419-10.177)	0.008	1.764 (0.396-7.861)	0.457
Primary tumor size	1.067 (0.893-1.275)	0.476		
Ki-67≥3%	1.597 (0.530-4.816)	0.406		0.259
NE	0.997 (0.994-0.999)	0.013	0.999 (0.996-1.001)	
E	0.966 (0.935-0.998)	0.035	0.986 (0.961-1.012)	0.292
DA	0.999 (0.998-1.001)	0.517		
SDHB mutation	12.778 (3.151-51.811)	0.000	5.334 (1.072-26.534)	0.041

EAPGL represents extra-adrenal pheochromocytoma. NE represents 24-h urinary norepinephrine; E represents 24-h urinary epinephrine; DA represents 24-h urinary dopamine; and SDHB represents succinate dehydrogenase subunit B.

### 3.4 Bone metastasis-free survival and overall survival

At the last follow-up examination, 5 of the 107 patients (4.7%) had died, and the median OS was not reached. Eighty-one of the 107 patients were diagnosed with postoperative BMs, and the median BMFS was 108 months (range, 3–308 months). Univariable analysis revealed that the factors significantly associated with shorter bone metastasis-free survival were EAPGL (HR, 3.701 [95% CI, 1.786–7.670]; *P* =0.000), lower 24h U-NE concentrations (HR, 0.998 [95% CI, 0.996–1.000]; *P* =0.029), and SDHB mutation (HR, 5.270 [95% CI, 0.190–0.088]; *P* =0.000). Multivariate Cox proportional hazards regression analysis revealed that SDHB mutation (HR, 3.376 [95% CI, 1.470–7.754]; *P* =0.004) played an independent prognostic role (**Table 7**).

**Table 7:** Univariate and multivariate Cox regression analyses of bone metastasis-free survival.

Variable	Univariable		Multivariable	
	*HR (95% CI)*	*P*	*HR (95% CI)*	*P*
Male	1.240 (0.610-2.523)	0.552		
Age	1.005 (0.976-1.035)	0.730		
EAPGL	3.701 (1.786-7.670)	0.000	2.105 (0.959-4.623)	0.064
Primary tumor size	1.074 (0.958-1.205)	0.222		
Ki-67≥3%	1.272 (0.584-2.769)	0.544		0.291
NE	0.998 (0.996-1.000)	0.029	0.999 (0.998-1.001)	
E	0.977 (0.952-1.002)	0.070		
DA	1.000 (0.999-1.001)	0.887		
SDHB mutation	5.270 (2.448-11.344)	0.000	3.376 (1.470-7.754)	0.004

EAPGL represents extra-adrenal pheochromocytoma. NE represents 24-h urinary norepinephrine; E represents 24-h urinary epinephrine; DA represents 24-h urinary dopamine; and SDHB represents succinate dehydrogenase subunit B.

**Figure 1:**
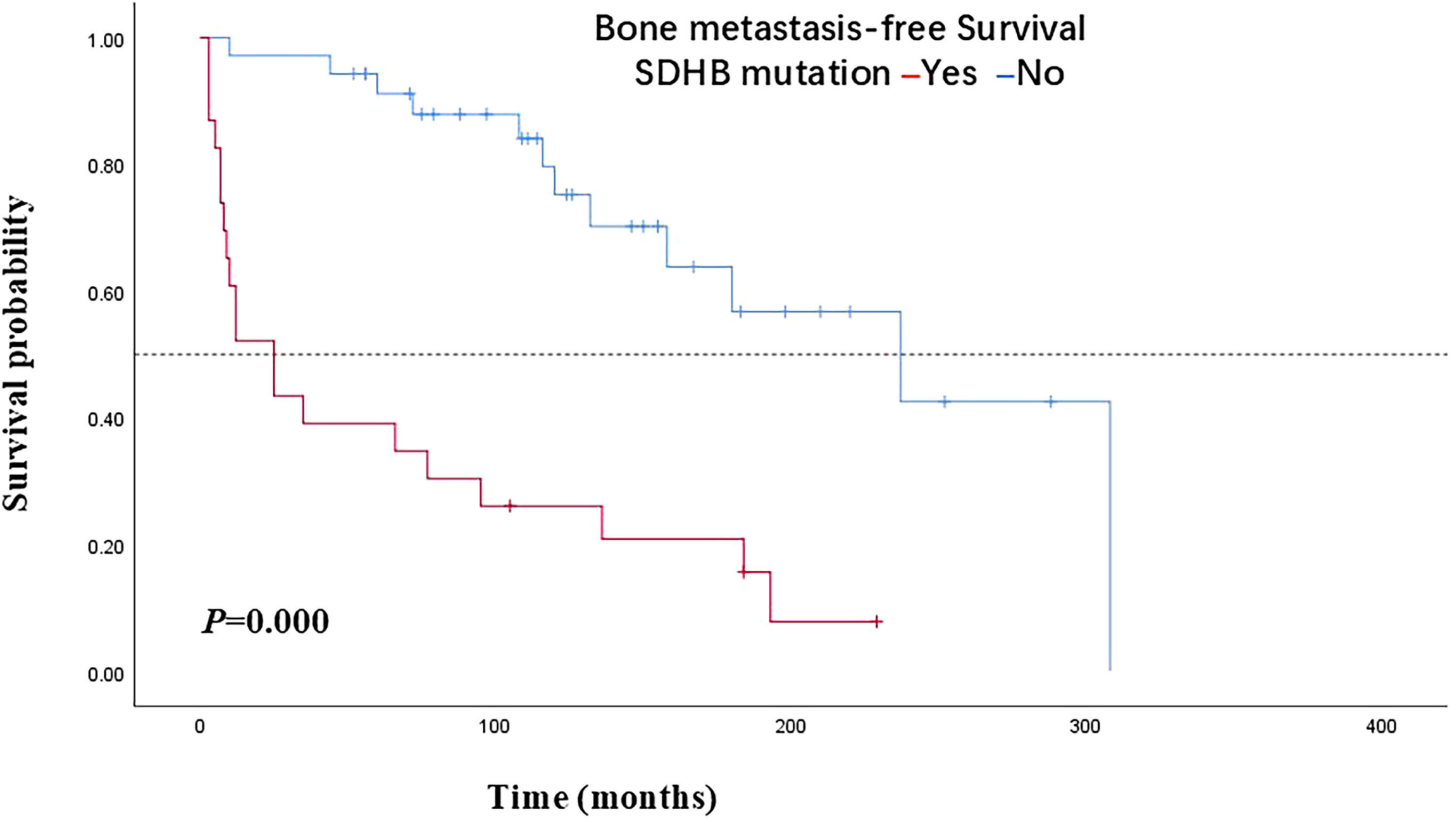
Bone metastasis-free survival in PPGL patients with SDHB mutations versus patients without SDHB mutations. PPGL, pheochromocytoma/paraganglioma; SDHB, succinate dehydrogenase subunit B.

**Figure 2:**
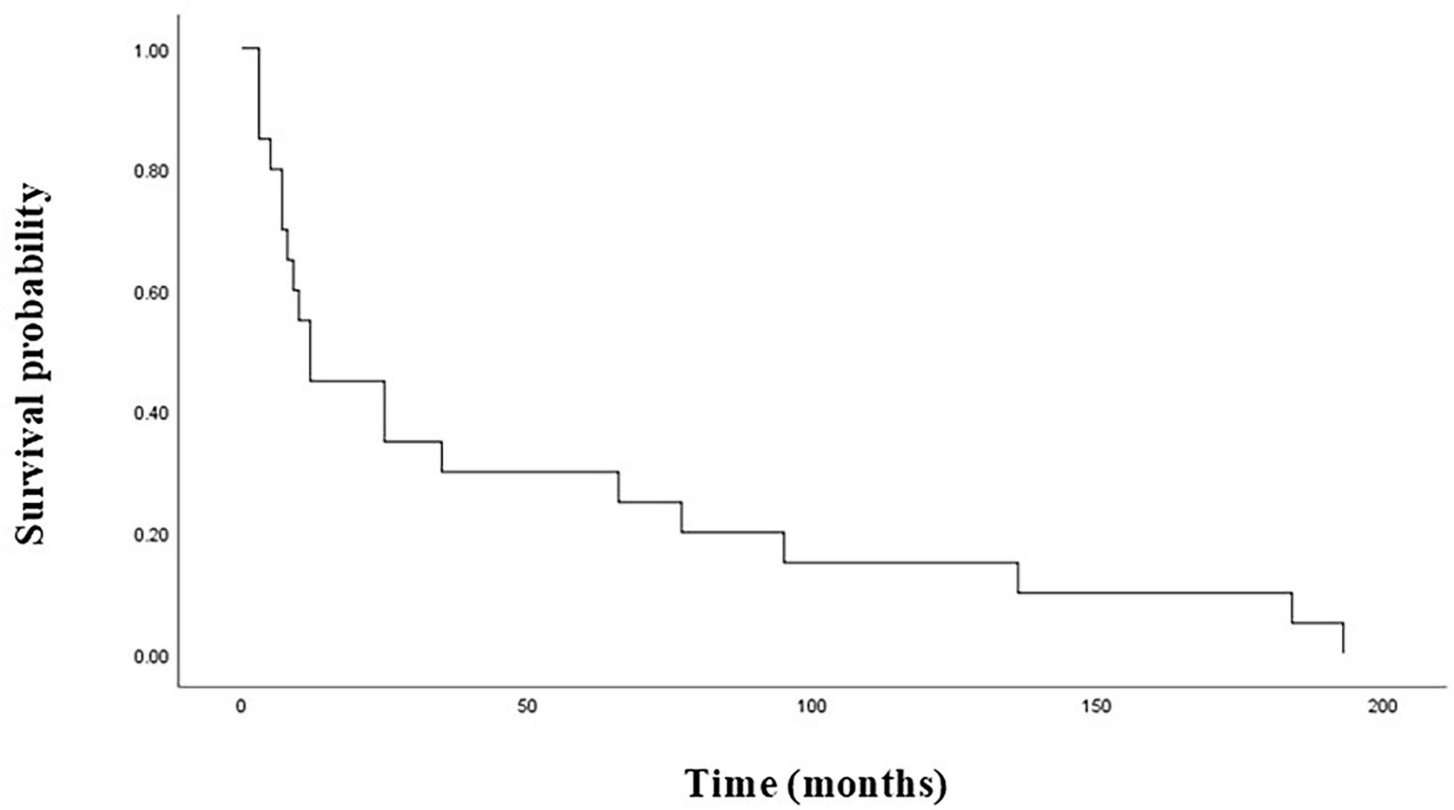
Bone metastasis-free survival in bone-metastatic PPGL patients with SDHB mutations. PPGL, pheochromocytoma/paraganglioma; SDHB, succinate dehydrogenase subunit B.

In terms of the 58 PPGLs, the median BMFS of PPGL patients with or without SDHB mutations was 25 months and 237 months, respectively, and the difference was statistically significant (*P*=0.000) (**Figure 1**). SDHB mutations were detected in 20 of the 32 bone-metastatic PPGLs, and further analysis revealed that the median BMFS of those patients was 12 months, significantly shorter than that of bone-metastatic patients without SDHB mutation (116 months) (**Figure 2**).

## 4 Discussion

Surgical intervention is typically the preferred treatment option for most PPGL patients, and some patients may develop postoperative recurrence or distant metastasis. Metastases in PPGLs are usually metachronous and occur after long latency following surgery for the primary tumor. Disease that is not localized in the usual regions of the PPGLs, such as lymph nodes and bone, is considered metastatic. In addition, metastases can be present in other organs, such as the liver and lungs, although they can originate as primary tumors. PPGLs should be managed according to tumor size, location, age at first diagnosis, presence of metastases, and especially underlying mutations in the era of precision medicine (7, 8). The incidence of metastatic PPGLs is extremely low, and there is a lack of high-level evidence from large-sample data on PPGLs with BM. The aim of this study was to address this gap by analyzing the clinical characteristics of patients with PPGLs who developed BM after primary tumor resection and to provide valuable insights into future clinical decisions concerning PPGLs.

Previous reports indicated that 9.5% of PPGLs had newly identified metastases during follow-up after initial diagnosis, and the median time between PPGL diagnosis and the detection of BM was reported to be 3.4 years. Long-term clinical observation revealed that most BMs progress slowly (9). Regular postoperative follow-up is crucial for all PPGLs because of the potential for metastasis. Imaging examinations, which involve both anatomical localization and functional imaging, play a vital role in monitoring disease recurrence and guiding treatment decisions on the basis of imaging results (10, 11). In this study, all 81 patients with PPGLs presented with their first BM during postoperative follow-up examinations, with a median time of 60 (ranging from 3 to 308) months, and most BMs occurred more than 5 years after surgery. Early BM may present as a single lesion, whereas in later stages, it can develop into widespread BMs throughout the body. Previous reports indicate that patients with PPGLs often develop multiple bone metastases, with 81% occurring in the spine, followed by the sacrum and pelvis (67%), long bones (49%), and skull (21%) (13). In our study, a total of 362 countable bone metastatic lesions were identified in 81 patients. The majority of patients (79.0%) presented with multiple bone metastases, and 61.7% of these bone-metastatic cases had concomitant metastases in other sites, such as the liver and lung. Among the 362 bone lesions, the most frequent site was the spine (63.3%), followed by the pelvis (18.5%), ribs, sternum, scapula and clavicle (10.2%), limbs (4.7%), and skull (3.3%).

We found that 44.4% of the patients with PPGLs presented skeletal complications, and pain due to BMs was the most frequently observed complication (83.3%), followed by spinal cord compression (25.0%), pathological fractures (13.9%), and hypercalcemia (5.6%). It has been reported that approximately 70%-80% of patients with BMs experience skeletal complications, and the proportions of the distribution of single complications were similar to our results, such as bone pain (33%-60%), fractures (8%-27%), spinal cord compression (20%-25%), and hypercalcemia (12%) (13, 14).

In clinical practice, some PPGL patient already have multiple metastases at the time of diagnosis, which may preclude the possibility of surgical intervention. Unfortunately, there is currently no clear and effective predictive factor for metastasis in PPGLs. Histological scoring systems such as PASS or GAPP are used to differentiate between benign and malignant tumors, but the process of distinguishing between the two remains challenging (4).

PPGLs are associated with potential genetic variations, and approximately 30% to 50% of PPGL patients have detectable underlying germline mutations (2, 3). Multiple studies have demonstrated that SDHB mutations are associated with an increased risk of metastatic disease (up to 60%) (8–12). In our study, 53.4% of the patients had gene mutations, 74.2% of which were in the SDHB gene. In a retrospective multicenter, multinational study, patients with SDHB mutations were more likely to develop bone metastasis (15). Similarly, our research confirmed that SDHB mutation was a risk factor for BM in PPGLs. Previous studies revealed that tumors occurring outside the adrenal gland were effective predictors of metastasis and that the primary tumor was larger in patients with metastases than in patients without metastatic disease (16, 17). Other studies have reported that positive staining for Ki-67 (≥3%) and catecholamine hypersecretion are associated with metastatic disease (18, 19). However, another study revealed that the above factors were not associated with the metastasis of PPGLs (20). Our study revealed no correlation between primary tumor site, tumor size, the Ki-67 index, the catecholamine level or bone metastasis in PPGLs. The differences in the results of the various studies might be attributed to the small sample size and relatively short follow-up time; the exact cause warrants further study.

Previous studies have shown that compared with patients with visceral metastases, bone-metastatic PPGL patients have a longer median survival period (12 years vs. 5–5.7 years), without a negative effect on survival (5, 13–15). At the last follow-up in our study, 5 of the 107 patients had died, of whom 2 had bone-metastatic PPGLs. The survival rate for patients with bone-metastatic PPGLs was high, and the median OS was not reached. However, our study revealed that the BMFS of patients with SDHB mutations was significantly lower than that of patients without SDHB mutations. As BMs did not adversely affect the survival of our patients, most patients presented skeletal complications for extended periods, resulting in significant declines in quality of life. Multidisciplinary comprehensive treatment approaches, increasingly used alongside primary tumor therapy, include local treatments (e.g., radiation therapy and orthopedic surgery), symptomatic bone pain management, and bone resorption inhibitors to inhibit osteolysis and prevent skeletal morbidity, thereby minimizing the impact of metastatic bone disease on physical function (15, 21–23). Therefore, it is crucial to assess the risk of bone metastasis in PPGL patients, thereby enabling early diagnosis and treatment to improve their quality of life.

At present, the use of MIBG radionuclide therapy for PPGL patients with locally progressive, uncontrollable, metastatic disease has demonstrated efficacy (24, 25). A study demonstrated that MIBG radionuclide therapy is associated with a reduced risk of death in bone-metastatic PPGLs (15). Additionally, peptide receptor-mediated radionuclide therapy (PRRT) has shown promising efficacy and safety in the treatment of metastatic PPGLs (26, 27). Furthermore, studies investigating the molecular signaling pathways and metabolic mechanisms of PPGLs have identified potential targets for personalized targeted drug therapy. Modification of specific signaling pathways, enzymes, and receptors has shown promising clinical efficacy (28, 29). In conclusion, therapeutic options based on molecular biomarkers are crucial for achieving precision medicine (PM) in patients with metastatic PPGL (30, 31).

However, our study has several limitations: a relatively small sample size, inherent selection biases and confounding factors because of its retrospective nature, and incomplete genetic testing data—largely attributable to the high cost of testing and the limited financial resources of some patients. These limitations should be considered when interpreting and generalizing the results; a multicenter cohort study with longer follow-up is warranted to overcome these shortcomings.

## 5 Conclusions

Given the growing number of PPGL-related genes discovered, genetic testing is now recommended for all PPGL patients. We emphasize that patients with SDHB mutations warrant early identification and regular monitoring due to their heightened risk of developing BMs. Timely assessment and management of BMs can significantly improve patients’ quality of life. In conclusion, a standardized yet personalized follow-up strategy is essential for monitoring disease progression, adapting treatment plans, and providing crucial information for familial counseling.

## Data Availability

The original contributions presented in the study are included in the article/supplementary material. Further inquiries can be directed to the corresponding authors.
